# Use of Minimal Residual Disease Status to Reduce Uncertainty in Estimating Long-term Survival Outcomes for Newly Diagnosed Multiple Myeloma Patients

**DOI:** 10.36469/001c.56072

**Published:** 2023-01-06

**Authors:** Naomi van Hest, Peter Morten, Keith Stubbs, Nicola Trevor

**Affiliations:** 1 Costello Medical Consulting, Cambridge, United Kingdom; 2 Janssen-Cilag Ltd, High Wycombe, United Kingdom

**Keywords:** multiple myeloma, minimal residual disease, PSM, landmark analysis, CASSIOPEIA, daratumumab, DBTd, uncertainty

## Abstract

**Background:** Demonstrating the cost-effectiveness of new treatments for multiple myeloma (MM) often relies on the extrapolation of overall survival (OS) trial data. This method can introduce uncertainty in long-term survival estimates if OS data are immature, as is often the case in newly diagnosed MM (NDMM). We explore the use of the relationship between minimal residual disease (MRD) status and OS to reduce uncertainty of long-term survival outcomes.

**Objectives:** To evaluate if uncertainty in long-term modeled outcomes in NDMM is reduced using a response-based partitioned survival model (PSM), whereby patients were categorized as MRD-positive or -negative, relative to a standard PSM, when OS data are immature.

**Methods:** Standard and response-based PSMs, estimating patient life-years (LYs) over a lifetime horizon, were developed for NDMM patients treated with bortezomib, thalidomide, and dexamethasone (BTd) with or without daratumumab as induction and consolidation therapy. In the standard PSM, LYs were determined by extrapolations from individual patient data from CASSIOPEIA. In the response-based PSM, survival was dependent on MRD status at the time of the response assessment via a landmark analysis. Cox-proportional hazard ratios from external sources and CASSIOPEIA informed the relationship for OS between MRD-positive and MRD-negative, and between patients receiving BTd and daratumumab plus BTd, respectively. Uncertainty was assessed by comparing LYs and OS extrapolations from deterministic and probabilistic analyses.

**Results:** This response-based PSM demonstrated reduced uncertainty in long-term survival outcomes compared with the standard PSM (range across extrapolations of 3.4 and 7.7 LYs for daratumumab plus BTd and BTd, respectively, vs 14.8 and 11.8 LYs for the standard PSM). It also estimated a narrower interquartile range of LYs in the probabilistic analyses for the majority of parametric extrapolations.

**Discussion:** Alternative methods to estimate long-term survival outcomes, such as a response-based PSM, can reduce uncertainty in modeling predictions around cost-effectiveness estimates for health technology assessment bodies and payers, thereby supporting faster market access for novel therapies with immature survival data.

**Conclusions:** Use of MRD status in a response-based PSM reduces uncertainty in modeling long-term survival in patients with NDMM and provides a greater number of clinically plausible extrapolations compared with a standard PSM.

## BACKGROUND

Multiple myeloma (MM) is a rare and incurable blood cancer with orphan disease designation in both the United States and Europe.^1,2^ The disease follows a relapsing-remitting course with almost all surviving MM patients eventually relapsing or becoming refractory to existing treatment options.^3–6^ With each relapse, it becomes increasingly difficult to induce deep and durable responses, leading to increased attrition rates.^7,8^ Therefore, it is important to use the most effective treatments as early as possible, as patients may not survive or be fit enough to receive treatment at later lines of therapy.

For patients with newly diagnosed MM (NDMM), induction therapy followed by autologous stem cell transplant (ASCT) with or without consolidation therapy represents the standard of care for patients fit enough to receive these interventions.^9,10^ Transplant-eligible NDMM patients have the best chance of achieving long-term sustained remission compared with other patients with MM.^11,12^ Despite this, survival outcomes in this population remain substantially lower than in the general population.^13^ This reflects, in part, the considerable heterogeneity in this patient population, with some patients relapsing early or not achieving a response to treatment, resulting in comparatively poorer outcomes.^14,15^ Therefore, there is still a substantial unmet need for more effective, well-tolerated treatment options in the frontline setting.

Overall survival (OS) and progression-free survival (PFS) are the standard, established endpoints used by regulatory agencies in their assessment of novel therapies for NDMM. However, for emerging therapies in earlier lines of treatment, the time frame needed to demonstrate statistically significant or clinically meaningful improvements in PFS alone in clinical trials is over 5 years.^16^

Long-term estimates of OS are needed as part of cost-effectiveness analyses, which are typically assessed over a lifelong time horizon. Therefore, cost-effectiveness models to inform these analyses, such as PSMs, rely on the extrapolation of survival data from clinical trials. Methods to extrapolate survival data are available, such as those described in Technical Support Document 14, published by the National Institute of Health and Care Excellence (NICE) Decision Support Unit (DSU).^17^ However, considerable uncertainty in long-term survival estimates is introduced if survival data are immature, which can hamper health technology assessment (HTA) decision-making. Recognizing the limitations of standard methods, the NICE DSU has published Technical Support Document 21, which details more flexible methods (eg, landmark analyses, among others) to model lifetime effects when hazard functions are complex.^13^ To effectively utilize this landmark method, a reliable marker of survival in NDMM to reduce uncertainty of long-term survival outcomes is required.

### Value of Minimal Residual Disease Status in Multiple Myeloma

Relapse in MM is due to cancerous cells that resist treatment and undergo clonal expansion and evolution, resulting in tumor repopulation. These remaining cells that contribute to relapse are known as minimal residual disease (MRD).^18,19^ The state of MRD negativity is one where no remaining clonal or subclonal cancerous cells can be detected using currently available measurement techniques. Patients who achieve this state have been shown to be less likely to relapse, with long-term disease control achieved for some patients. In recent years, the International Myeloma Working Group has recognized MRD as the most sensitive measure of response assessment for MM.^18,19^

The significance of MRD status and its relationship with long-term survival outcomes in patients with NDMM have been established in the literature through systematic literature reviews (SLRs) and meta-analyses.^20–22^ The results of these studies have established the clinical utility of MRD assessment as an important marker of survival and could therefore be considered when estimating long-term outcomes when survival data are immature. Compared with other outcomes, MRD has also been shown to be superior and the most relevant predictor of clinical outcomes in MM via multivariate analyses.^19,21,23–26^ Furthermore, MRD status was recently used as the primary endpoint in the ongoing CEPHEUS trial for NDMM.^27^ Recently, the European Medicines Agency considered there to be uncertainty in the quantitative, predictive association between MRD negativity and clinical endpoints like OS and PFS. However, it should be noted that assessment was made considering the use of MRD status as a primary endpoint in clinical trials to be used for regulatory decision-making. The correlation between MRD status and survival has been well established in the literature and the European Medicines Agency acknowledged this correlation at the patient level.^28^

A prior study by Yamamoto et al incorporated MRD status as a predictive marker in a cost-effectiveness analysis of daratumumab in NDMM used a Markov model structure.^29^ The current study differs from the approach of Yamamoto et al as it incorporates external data, whereas the previous study relied only on trial data. Secondly, this study provides a comparison between a standard partitioned survival model (PSM) and a PSM incorporating MRD status to assess the impact on uncertainty. The previous study presented cost-effectiveness results alone and did not provide this comparison.

### Introduction to Daratumumab

Daratumumab is a first-in-class, fully human IgG1**κ** monoclonal antibody that binds to CD38, a cell-surface glycoprotein found on many immune cells, including white blood cells.^30,31^ Among others, daratumumab is indicated in combination with bortezomib, thalidomide, and dexamethasone (DBTd) for the treatment of adult patients with NDMM who are eligible for ASCT.^22^

Evidence of the clinical effectiveness of DBTd was demonstrated in CASSIOPEIA, a pivotal randomized, open-label, active-controlled, parallel-group, multicenter phase 3 study.^32^ The trial included 1085 patients, with a median follow-up of 29.2 months. The treatment phase was split into 2 parts; only data from part 1 were used in this study, which reflects the current approved use of daratumumab in this indication. In part 1, patients were randomized to receive four 28-day cycles of induction therapy with DBTd or bortezomib, thalidomide, and dexamethasone (BTd) prior to ASCT, followed by two 28-day cycles of consolidation therapy with DBTd or BTd. The primary endpoint in part 1 was the proportion of patients achieving stringent complete response. Key secondary endpoints included PFS, OS, and rate of MRD negativity. Minimal residual disease negativity was measured in CASSIOPEIA at 100 days post-ASCT, to assess efficacy following induction, ASCT and consolidation therapy.^32^ As measured at postconsolidation, a significantly higher proportion of patients were MRD-negative (MRD-) measured using multiparametric flow cytometry when treated with DBTd compared with BTd alone at a threshold of 1 tumor cell per 10^-5^ white cells (64% vs 44%; *P* < .0001).^32^ Survival data from CASSIOPEIA are, however, considered immature as only a small proportion of patients experienced an OS event in the BTd and DBTd arms at a median follow-up of 29.2 months (8.9% and 4.8%, respectively).^33^

### Study Objectives

The objective of this study was to assess the uncertainty in modeled outcomes associated with the use of a response-based PSM, relative to a standard PSM, when survival data are immature. Using this approach, the study also aimed to evaluate the suitability of MRD status as a marker for modeling long-term survival outcomes in NDMM by assessing the clinical plausibility of results from each modeling approach.

## METHODS

### Setting

A de novo PSM, which estimated patient life-years (LYs), was developed in Microsoft Excel for patients with NDMM who are eligible for ASCT. To reflect the CASSIOPEIA trial, patients were modeled to receive induction therapy, with either DBTd or BTd, followed by ASCT and consolidation therapy (as per their induction therapy).

### Model Structure

In line with guidance from NICE DSU 19, both a PSM and state transition were developed initially. The PSM structure was deemed more appropriate as it allows intuitive incorporation of PFS and OS data from the CASSIOPEIA trial, and preliminary analyses indicated results would be comparable. Therefore, a PSM structure was pursued further.^34^

A standard PSM and response-based PSM were independently explored. In the standard PSM, the occupancy of health states over time was derived from survival curves directly extrapolated from individual patient data (IPD). In the response-based PSM, long-term outcomes were modeled to be dependent on whether patients had achieved a response to treatment, with “responders” defined as patients who were MRD- at a particular time point. Compared with the standard PSM, this approach explicitly acknowledges, and can better account for, underlying patient heterogeneity between responders and nonresponders.

To mitigate against the effect of immortal time bias (ie, the need for patients to live long enough to experience an event), a “landmark” approach was taken in the response-based PSM in line with NICE DSU guidance.^13^ The selected landmark time point was 100 days post-ASCT, to align with the timing of the MRD response assessment in the CASSIOPEIA trial.^32^ This time point is frequently chosen in MM trials because it is considered to be sufficiently long to capture the benefit of consolidation while being short enough to ensure few events were censored.^21,35^ Survival after this time point was modeled depending on MRD status at the time of the response assessment, with separate survival inputs used for patients with MRD- and MRD-positive (MRD+) status. Prior to the landmark time point, patients were modeled based on the observed survival data from CASSIOPEIA.^32^

**Figure 1.**
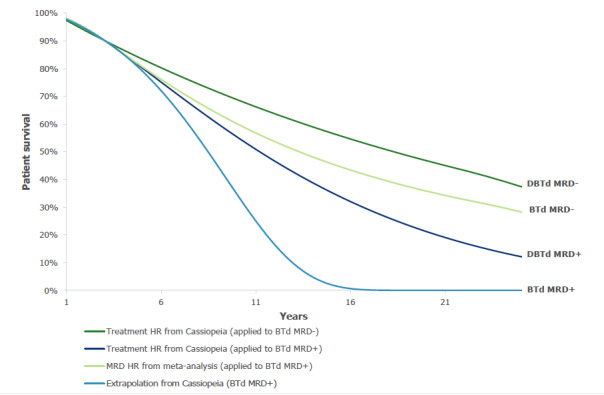
Approach to Modeling Survival by Response and Treatment Arm for the Response-based PSM Survival presented is illustrative only and not expected to reflect the absolute or relative survival of these treatment and response curves. Abbreviations: BTd, bortezomib, thalidomide and dexamethasone; DBTd, daratumumab, bortezomib, thalidomide and dexamethasone; HR, hazard ratio; MRD+/-, minimal residual disease positive/negative; PSM, partitioned survival model.

**Figure 2.**
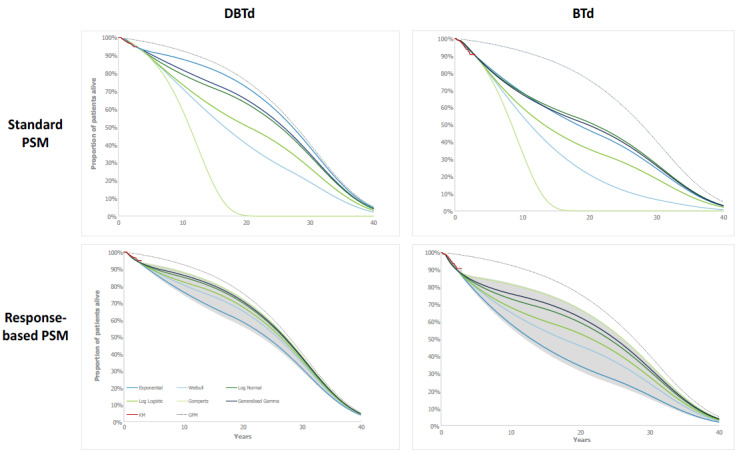
Long-term Modeled Extrapolations by Treatment and Model Structure For the response-based PSM, curve choices refer to the extrapolation of MRD+ BTd patients; HRs are then applied to this extrapolation to generate survival estimates for the full BTd cohort and the DBTd cohort. Areas shaded grey show where the curves sit when the HRs are varied across the 95% CIs. Abbreviations: BTd, bortezomib, thalidomide and dexamethasone; DBTd, daratumumab, bortezomib, thalidomide and dexamethasone; GPM, general population mortality; HR, hazard ratio; PSM, partitioned survival model.

The following approach was taken for modeling post-landmark outcomes in the response-based PSM (Figure 1). For the BTd MRD+ curve, IPD from CASSIOPEIA for this cohort of patients were extrapolated. Of all cohorts of interest, this was the most mature data set from CASSIOPEIA and was used as a reference curve. For the BTd MRD- curve, a Cox-proportional hazard ratio (HR) for MRD- relative to MRD+ patients was applied to the BTd MRD+ survival curve, utilizing long-term data for NDMM transplant-eligible patients from the published SLRs and meta-analyses that assessed the relationship between MRD status and survival outcomes.^20–22^ For the DBTd MRD+ and DBTd MRD- curves, MRD-positive- and MRD-negative-specific Cox-proportional HRs for DBTd vs BTd were applied to the corresponding BTd survival curves, utilizing information on the DBTd treatment effect derived from the landmark analysis of the CASSIOPEIA trial. The resultant extrapolations for each treatment (Figure 2) were validated against the observed Kaplan-Meier data and other published data (Table 1). The sources of these data and inputs are described below.

### Clinical Inputs

Survival curves for DBTd and BTd were based on IPD from the intention-to-treat population of the CASSIOPEIA trial.^32^ Where appropriate, survival extrapolations were conducted in line with NICE DSU guidance,^17^ considering the full range of recommended parametric distributions (exponential, Weibull, log logistic, log normal, Gompertz, and generalized gamma). The appropriateness of modeling survival via the use of HRs was dependent on the assumption of proportional hazards. This was assessed and confirmed for all cases via inspection of log-cumulative hazard Schoenfeld residual plots, as per NICE DSU guidance (**Supplementary Figure S1**).^17^

The HR between MRD- and MRD+ applied to the reference curve was based on an SLR/meta-analysis conducted by Munshi et al, which identified 143 publications reporting survival outcomes in MM by MRD status.^20–22^ The results of the SLR were subsequently adapted to limit the analysis to patients in whom transplant was performed and MRD was assessed at 100 days post-SCT, and who were not treated with DBTd. Therefore, the DBTd arm of the CASSIOPEIA trial was excluded. This ensured that the included HRs aligned to the patient population in the model. As a result, 7 OS HRs informed the value implemented in the model. The clinical inputs used in the response-based PSM are shown in Table 1.

**Table 1.** Clinical Inputs for the Response-Based PSM Model

**Model Input**	**Parameter Value**	**Source**	**Model Application**
**MRD-negative patients, % (95% CI)^a^**			
DBTd	63.7 (59.5- 67.8)^b^	CASSIOPEIA (ITT population)	To determine proportion of patients achieving MRD negativity
BTd	43.5 (39.3-47.8)^b^	CASSIOPEIA (ITT population)	
**HR applied to OS parametric extrapolation, HR (95% CI)**			
MRD- vs MRD+	0.60 (0.50-0.72)^c^	Analysis conducted on Munshi et al^20^	To estimate survival outcomes for MRD- BTd patients (applied to BTd MRD+)
DBTd MRD+ vs BTd MRD+	0.73 (0.40-1.35)	CASSIOPEIA (landmark analysis)	To estimate survival outcomes for MRD+ DBTd patients (applied to BTd MRD+)
DBTd MRD- vs BTd MRD-	0.41 (0.16-1.04)	CASSIOPEIA (landmark analysis)	To estimate survival outcomes for MRD- DBTd patients (applied to BTd MRD-)

HR, hazard ratio; ITT, intention to treat; MRD+/-, minimal residual disease positive/negative; OS, overall survival.

^a^The proportion of patients achieving postconsolidation MRD negativity in the model was based on that in CASSIOPEIA, with a sensitivity threshold of 10^-5^ for MRD.

^b^DBTd vs BTd: *P* < .0001.

^c^The HR used in the model differs slightly from the HR reported in Munshi et al^20^ due to the exclusion of the DBTd arm of the CASSIOPEIA trial and studies measuring MRD at 100-days post–autologous stem cell transplant from the analysis.

The risk of mortality for transplant-eligible patients with NDMM is expected to be higher than those of the general population when matched for age and gender. Therefore, to ensure that the extrapolated OS did not exceed that of the general population, age- and gender-matched general population mortality (GPM) in England was used in any cycle where the predicted probability of death was lower than GPM.

### Model Outcomes and Validation

The model estimated LYs for the total patient population over a lifetime horizon (43 years, based on a mean age of 57 years from CASSIOPEIA and assuming all patients were dead at 100 years). Long-term OS extrapolations were assessed using a combination of statistical goodness-of-fit criteria (see **Supplementary Table S1**), visual inspection, and clinical expert opinion.^17^

For the base case analysis, the choice of extrapolation was informed by feedback from UK clinicians on the long-term survival outcomes expected in clinical practice. In the standard PSM, a Weibull and log logistic curve was selected for BTd and DBTd, respectively. For the response-based PSM, an exponential curve was selected for BTd MRD+.

The survival outcomes predicted by the response-based PSM, which incorporated both MRD+ and MRD- patients, and the standard PSM were then compared against: (1) observed data from the CASSIOPEIA trial (DBTd and BTd)^32^; (2) observed data from the GIMEMA trial,^36^ a phase 3 randomized controlled trial (n = 474; median follow-up, 10.3 years), which compared BTd with thalidomide and dexamethasone as induction and consolidation therapy after double ASCT for NDMM; and (3) data from National Health Service (NHS) Digital datasets on real-world survival outcomes for 1049 patients in the United Kingdom who received first-line BTd.^37^

### Evaluation of Model Uncertainty

To evaluate uncertainty in model estimates, mean and median LYs for each of the 6 parametric extrapolations were evaluated. To explore uncertainty within each parametric extrapolation, probabilistic sensitivity analyses (PSAs) were conducted. A total of 2000 simulations were conducted, based on the number of simulations required for stabilization of the model results, per parametric extrapolation and model structure, in which the individual parameters informing each extrapolation were varied according to a normal distribution (Cholesky decomposition). In the response-based PSM, the HRs (log normal distribution), and the MRD- rate (beta distribution) were also sampled.

## RESULTS

### External Validation

With the selected base case extrapolations, patients were estimated to live an average of 14 and 21 years for BTd and DBTd, respectively, in the standard PSM and 16 and 22 years, respectively, in the response-based PSM (Table 2). Comparison of the base case LYs estimated by both models to the observed data demonstrated that both generate clinically plausible results: the plausibility of model estimates was supported both in the short- and longer-term by comparing with data from the clinical trials and real-world settings (Table 3). The base case extrapolations in both the standard and response-based PSM, and across both treatment arms, produced closely matching estimates of OS compared with the observed data from the CASSIOPEIA trial, based on 2 years of follow-up.

**Table 2.** Estimated Mean Life-Years by Treatment and Model Structure

**Extrapolation Curve**	**Standard PSM (LYs)**				**Response-Based PSM (LYs)^a^**			
	**DBTd**		**BTd**		**DBTd**		**BTd**	
	**Mean**	**Median**	**Mean**	**Median**	**Mean**	**Median**	**Mean**	**Median**
Exponential	24.18	26.52	19.60	18.77	22.49	24.68	16.36	13.31
Weibull	18.78	17.02	13.58	11.46	23.98	26.52	19.01	18.08
Log normal	23.58	25.99	20.31	21.38	25.21	27.60	22.00	24.92
Log logistic	20.97	20.85	16.94	13.62	24.56	27.06	20.43	22.31
Gompertz	11.09	11.42	8.48	8.69	25.87	27.98	24.04	26.92
Generalized gamma	25.90	27.98	19.95	20.38	25.50	27.75	22.78	25.85
**Range^b^**	**14.81**	**16.56**	**11.84**	**12.69**	**3.37**	**3.30**	**7.68**	**13.62**

Abbreviations: BTd, bortezomib, thalidomide, and dexamethasone; DBTd, daratumumab, bortezomib, thalidomide and dexamethasone; LY, life-year; MRD, minimal residual disease; NHS, National Health Service; PSM, partitioned survival model.

^a^Curve choices refer to the extrapolation of MRD-positive BTd patients; hazard ratios are then applied to this extrapolation to generate survival estimates for the full BTd cohort and the DBTd cohort.

^b^Ranges have been calculated from unrounded data and therefore may differ from the range of the values presented. Blue cells indicate the curves that were chosen for the base case extrapolations.

**Table 3.** Validation of Base Case Model Outcomes (Percentage of Patients Alive)

**Data Source**	**Time (mo)**					
	**12**	**18**	**24**	**36**	**60**	**120**
DBTd						
Standard PSM – log logistic	99	98	97	95	89	75
Response-based PSM – exponential (BTd MRD+)	98	97	95	93	88	77
CASSIOPEIA^32^	98	98	97	—	—	—
BTd						
Standard PSM – Weibull	98	97	95	90	81	57
Response-based PSM – exponential (BTd MRD+)	97	94	92	87	78	60
CASSIOPEIA^32^	98	95	93	—	—	—
GIMEMA^36^	97	93	91	86	79	60
NHS Digital cohort BTd as first-line treatment (ASCT-positive)^37^	98	96	93	88	—	—

Abbreviations: ASCT, autologous stem cell transplant; BTd, bortezomib, thalidomide, and dexamethasone; DBTd, daratumumab, bortezomib, thalidomide, and dexamethasone; HR, hazard ratio; MRD+, minimal residual disease–positive; NDMM, newly diagnosed multiple myeloma; PSM, partitioned survival model.

In addition, the base case extrapolations for BTd in both the standard and response-based PSM showed reasonably similar estimates of longer-term OS compared with observed data from the GIMEMA trial, based on 10 years of follow-up, and the observed real-world survival outcomes from NHS Digital, based on 3 years of follow-up.

### Uncertainty of Long-term Outcomes

In the standard PSM, the high level of divergence in long-term estimates across different parametric extrapolations for both the DBTd and BTd treatment arms reflects the immaturity of the OS data and resultant uncertainty. In comparison, the divergence in extrapolations was found to be substantially reduced in the response-based PSM (Figure 2). This is demonstrated by the mean LYs estimated by the different extrapolation curves across the lifetime time horizon in the standard PSM, which had a range (min-max) of 14.8 (11.1-25.9) and 11.8 (8.5-20.3) LYs for DBTd and BTd, respectively, compared with 3.4 (22.5-25.9) and 7.7 (16.4-4.0) LYs for the response-based PSM (Table 2). This trend remains when removing any clinically implausible extrapolations (ie, Gompertz for DBTd and BTd in the standard PSM; Figure 2). For DBTd in the standard PSM, the range decreased from 14.8 to 7.1 LYs when excluding the Gompertz curve, which is still greater than the range observed for DBTd in the response-based PSM of 3.4 LYs. For BTd, the range decreases from 11.8 to 6.7 LYs in the standard PSM, comparable to the range observed in the response-based PSM of 7.7 LYs.

**Figure 3.**
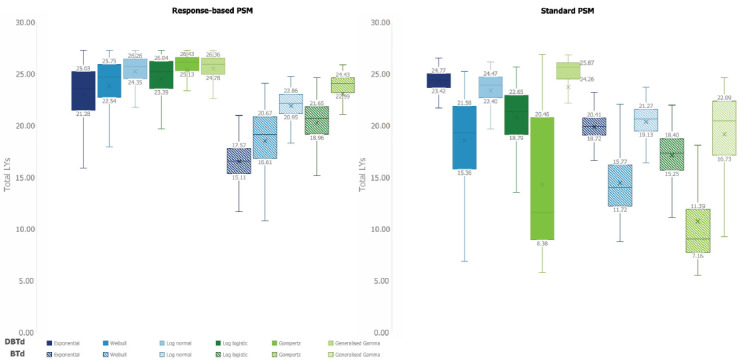
Mean Life-Years Estimated by 2000 Simulations, by Treatment and Model Structure Horizontal lines within the box indicate median LYs; X denotes mean LYs. For the response-based PSM, curve choices refer to the extrapolation of MRD+ BTd patients; hazard ratios are then applied to this extrapolation to generate survival estimates for the full BTd cohort and the DBTd cohort. For the standard PSM, curve choices refer to the extrapolation of DBTd and BTd patients directly from the CASSIOPEIA trial. Abbreviations: BTd, bortezomib, thalidomide and dexamethasone; DBTd, daratumumab, bortezomib, thalidomide and dexamethasone; LY, life-year; PSM, partitioned survival model.

These findings were supported by probabilistic simulations; the range of mean LYs estimated in the PSA from different parametric extrapolations is narrower in the response-based PSM than the standard PSM (**Figure 3**). Additionally, the probabilistic analyses demonstrated that uncertainty of LY estimates *within* a given parametric extrapolation (as indicated by the interquartile range [IQR] and range) was also reduced in the response-based PSM relative to the standard PSM for most extrapolations (**Figure 3; Supplementary Table 2**).

Although the uncertainty of the response-based PSM estimates was lower than that of the standard PSM between and within parametric extrapolations for both the DBTd and BTd arms, the reduction in uncertainty associated with the response-based model was greater for DBTd (**Figures 2 and 3**).

## DISCUSSION

### Interpretation of Results

The results demonstrated that this response-based PSM was associated with reduced uncertainty in long-term survival outcomes compared with the standard PSM, illustrated by the lower divergence in OS estimates and mean LYs predicted across different parametric extrapolations in the response-based PSM. This trend remained when excluding the clinically implausible extrapolations. The reduced uncertainty associated with this response-based PSM was further supported by the smaller range of LYs estimated for a given parametric model in the PSA, which was true for all parametric extrapolations across both treatment arms, with the exception of the exponential curve. The finding was particularly pronounced for DBTd due to the relative immaturity of the data used in the standard PSM for this treatment arm.

As such, the results support use of a response-based PSM, which in this case better accounts for underlying patient heterogeneity vs the standard PSM by modeling patients by MRD status. This approach also facilitates the incorporation of external data with longer follow-up and a more precise pooled estimate of effect, decreasing reliance on direct extrapolation of less mature survival data (eg, MRD- patients).

Despite the high level of divergence in long-term estimates across different parametric extrapolations for both DBTd and BTd, both the standard and response-based PSMs could generate plausible estimates of LYs compared with long-term survival estimates from GIMEMA and observed data from CASSIOPEIA. However, the reduced divergence of extrapolations in the response-based PSM provided a greater number of extrapolations that were clinically plausible. This demonstrates that, regardless of the approach taken, there is a need to explore multiple extrapolations and assess the clinical plausibility of extrapolations using external sources of validation and clinical expert opinion.

Within the response-based PSM approach, BTd showed more divergence in long-term OS estimates and a larger range of probabilistic LYs for the majority of extrapolations than DBTd. As the response-based PSM for DBTd includes all the same parameters as BTd, with the addition of 2 HRs applied to the BTd arm (associated with additional uncertainty), the increased divergence for BTd vs DBTd may appear counterintuitive as one may expect the uncertainty to be similar or increased across all extrapolations. This discrepancy is likely because of the GPM limit applied as a cap to OS, which has a greater influence on DBTd than BTd (in both model structures), given the improved survival of DBTd. This is exemplified by the shape of the response-based extrapolations, whereby the shapes of the DBTd curves were visually more similar to each other and the GPM curve than was seen for BTd. Additionally, the upper bounds of the DBTd IQRs on the response-based PSM were all tightly aligned, suggesting that there is an upper limit in the mean predicted LYs. Applying the GPM cap on extrapolations prevents implausible extrapolations whereby patients treated with DBTd are predicted to live beyond the general population, which would not be accounted for in the extrapolations of observed data. As a result, the uncertainty in long-term survival associated with DBTd was less than BTd, despite having 2 additional parameters associated with uncertainty.

Within each model structure, particularly the standard PSM, the level of uncertainty associated with each curve changed considerably. Depending on the hazard of the observed data, different curves are associated with different levels of uncertainty in long-term survival estimates.^38^ This is also partially explained by the GPM cap. For example, extrapolations with the smallest IQR in the standard PSM (exponential and log normal for both DBTd and BTd) had the most optimistic OS extrapolations, which were therefore more influenced by the GPM cap, limiting the extent to which their long-term extrapolations could vary. In comparison, extrapolations that had lower OS estimates (eg, Gompertz and Weibull for both DBTd and BTd) showed larger IQRs, reflective of more uncertainty.

It was also noted that when comparing between model structures, different extrapolations for the same treatment arms had different levels of associated uncertainty. This variability was expected given the underlying observed data used to inform the long-term survival estimates for each treatment were different between model structures.

### Strengths and Limitations

A key strength of the study was the demonstration of uncertainty through several means: namely, by demonstrating less divergence in the extrapolations for OS and smaller ranges in the predicted mean LYs, as well as demonstrating reduced uncertainty within each extrapolation method through probabilistic simulations. Furthermore, the comparison of model estimates against the observed data from CASSIOPEIA and a range of external data sources with long-term follow-up was critical to validate the survival estimates from each model structure.^32^

Both models were developed using IPD from the same source (CASSIOPEIA), supporting comparability of results and avoiding reliance on simulated IPD or aggregate data. The response-based PSM was developed in line with NICE DSU guidance for landmark analyses and leveraged a clinically meaningful landmark time point at which to assess MRD status. A major strength of utilizing a response-based model structure is that it acknowledges underlying patient heterogeneity and facilitates incorporation of external data with longer follow-up in the form of a pooled estimate, to inform the relationship between MRD status and OS.^20^ The response-based model structure was recently accepted by both NICE and the Scottish Medicines Consortium as part of the HTA process for daratumumab in this indication.^39,40^

A potential limitation of this analysis is how the GPM cap was implemented in the model. As noted in the methods, the survival hazard was set to the minimum of the GPM and the hazard of the OS extrapolation. Recently, alternative methods for incorporating GPM into survival extrapolations, such as incorporating GPM hazards in the log-link function, have come to light, which might influence survival estimates.^13,41^ As such, exploring alternative methods to incorporate GPM into this type of analysis requires further investigation. A second limitation of this analysis was that GPM was not sampled in the PSA. Therefore, any associated uncertainty in the GPM would not have been accounted for. However, since the GPM comes from population-level data (ie, the Office for National Statistics), it is not expected to be associated with parameter uncertainty and is therefore not typically included in probabilistic simulations.^42^

In taking a response-based modeling approach, the limitations associated with landmark analyses also apply.^13^ For example, the choice of MRD assessment time point may influence the analysis, and the reliability of the long-term survival data for each responder category can impact uncertainty. Splitting samples by response can also increase uncertainty in extrapolated estimates due to the smaller patient populations (ie, the BTD MRD+ population). We also acknowledge the appropriateness of modeling survival via the use of HRs is dependent on the assumption of proportional hazards over time. For the duration of the trial, this was assessed and confirmed via inspection of log-cumulative hazard plots (see **Supplementary Figure S1**) and was assumed to be maintained for the modeled time horizon. A further limitation of this model structure is that MRD status was assessed at a single time point (100 days post-ASCT). While recent relapsed/refractory MM studies indicate that sustained MRD negativity for 12 or more months translates to improved long-term outcomes, reported data on the significance of durable MRD response in the front-line setting is limited.^43^

### Implications

This paper demonstrates an alternative approach to long-term survival modeling for patients with NDMM following ASCT that reduces uncertainty compared with a standard PSM, by incorporating MRD status and external data in a response-based PSM framework, avoiding the need to fit standard parametric extrapolations to immature survival data. This alternative approach to cost-effective modeling in NDMM may have wider implications for modeling survival. For example, this response-based approach can be used to reduce uncertainty in long-term survival estimates in other disease areas, where (1) there are clear response categories with a validated relationship between response and survival and (2) mature survival data are not available at the time of drug assessment. By extension, this reduces uncertainty in cost-effectiveness results assessed by HTA bodies for decision-making, enabling faster access to novel treatments. To allow the use of this approach, drug manufacturers would benefit from ensuring the necessary data are collected when designing clinical trials (eg, defining a clinically relevant landmark time point and including a response assessment).

This response-based model produced more clinically plausible results than the standard PSM, aligning with the stronger clinical rationale underpinning the modeling approach that better captures patient heterogeneity. As targeted treatments, such as precision medicine and advanced therapy medicinal products, become more common and widely used, the use of models founded in biological plausibility in the face of immature data will be vital for estimating long-term outcomes and, ultimately, healthcare decision-making.

Furthermore, a response-based modeling approach provides a framework to assess and explore alternative pricing mechanisms, such as outcomes-based pricing.

## CONCLUSIONS

In disease areas with a relatively good prognosis, such as NDMM, estimating long-term survival for novel therapies is often challenging, as immature data can lead to considerable uncertainty in long-term survival estimates. Systematic literature reviews and meta-analyses have established MRD status as the most sensitive outcome in NDMM and supported its use as a surrogate for PFS and OS.^20–22^

This paper presents an alternative approach for estimating long-term survival in NDMM, leveraging MRD status via a landmark analysis with external data to inform the relationship between MRD status and OS. This approach demonstrates that the use of MRD status in a response-based PSM reduces uncertainty in modeling long-term survival in patients with NDMM who are eligible for ASCT by providing a greater number of clinically plausible extrapolations compared with a standard PSM. By reducing uncertainty in modeling predictions, the method presented here can support faster market access for patients to more effective medicines by reducing decision-making uncertainty around cost-effectiveness estimates for HTA bodies and payers. This method has potential to be applied in other disease areas with immature survival data and a suitable marker for survival.

### Author Contributions

N.v.H. participated in the conception, design and development of the economic model, substantial contributions to analysis and interpretation of the data and drafting of the manuscript. K.S. participated in the conception, design and development of the economic model, acquisition of data, substantial contributions to analysis and interpretation of the data and drafting of the manuscript. P.M. participated in the conception, design, and development of the economic model, substantial contributions to analysis and interpretation of the data, and drafting of the manuscript. N.T. participated in the conception, design and development of the economic model, acquisition of data, substantial contributions to analysis and interpretation of the data, and drafting of the manuscript.

### Disclosures

N.v.H. is an employee of Costello Medical Consulting; P.M. was an employee of Costello Medical Consulting at the time of this research, and K.S. and N.T. are employees of Janssen-Cilag Ltd.

## Supplementary Material

Online Supplementary Material
