# Supplementary material for: Use of Minimal Residual Disease Status to Reduce Uncertainty in Estimating Long-term Survival Outcomes for Newly Diagnosed Multiple Myeloma Patients
Source: J Health Econ Outcomes Res. 2023 Jan 6;10(1):1–9. doi: 10.36469/001c.56072 (PMC9826714; doi:10.36469/001c.56072)
Supplement: Online Supplementary Material [file jheor_2023_10_1_56072_132805.pdf]

Journal of Health Economics and Outcomes Research

Online Supplementary Material

Use of Minimal Residual Disease Status to Reduce Uncertainty in Estimating Long-term Survival Outcomes for Newly Diagnosed Multiple Myeloma Patients. *JHEOR*. **2023;10(1):1-9.** doi:10.36469/jheor.2023.56072

Table S1: Statistical Goodness of Fit by Treatment and Model Structure

Figure S1: Log-Cumulative Hazard and Schoenfeld Residual Plots

Table S2: Average Mean, Median, and Interquartile Ranges Estimated by 2000 Simulations, by Treatment and Model Structure

This supplementary material has been provided by the authors to give readers additional information about their work.

This is an open-access article distributed under the terms of the Creative Commons Attribution 4.0 International License (CCBY-4.0). View this license's legal deed at http://creativecommons.org/licenses/by/4.0 and legal code at http://creativecommons.org/licenses/by/4.0/legalcode for more information.

Table S1. Statistical Goodness of Fit by Treatment and Model Structure

Extrapolation Curve	Standard PSM (OS)				Response-Based PSM (OS)	
	DBTd		BTd		BTd MRD+	
	AIC	BIC	AIC	BIC	AIC	BIC
Exponential	391.54	395.84	659.73	664.03	404.29	407.96
Weibull	390.77	399.36	657.55	666.14	405.48	412.82
Log normal	389.09	397.69	656.68	665.27	403.70	411.04
Log logistic	390.65	399.24	657.29	665.88	405.11	412.45
Gompertz	392.69	401.28	659.90	668.49	403.36	410.70
Generalized gamma	388.15	401.04	658.67	671.56	405.44	416.45

Blue cells indicate the curves that were chosen for the base case extrapolations.

Abbreviations: AIC, Akaike information criterion; BIC, Bayesian information criterion; BTd, bortezomib, thalidomide, and dexamethasone; DBTd, daratumumab, bortezomib, thalidomide, and dexamethasone; MSD, minimal residual disease; OS, overall survival; PSM, partitioned survival model.

Figure S1. Log-Cumulative Hazard and Schoenfeld Residual Plots

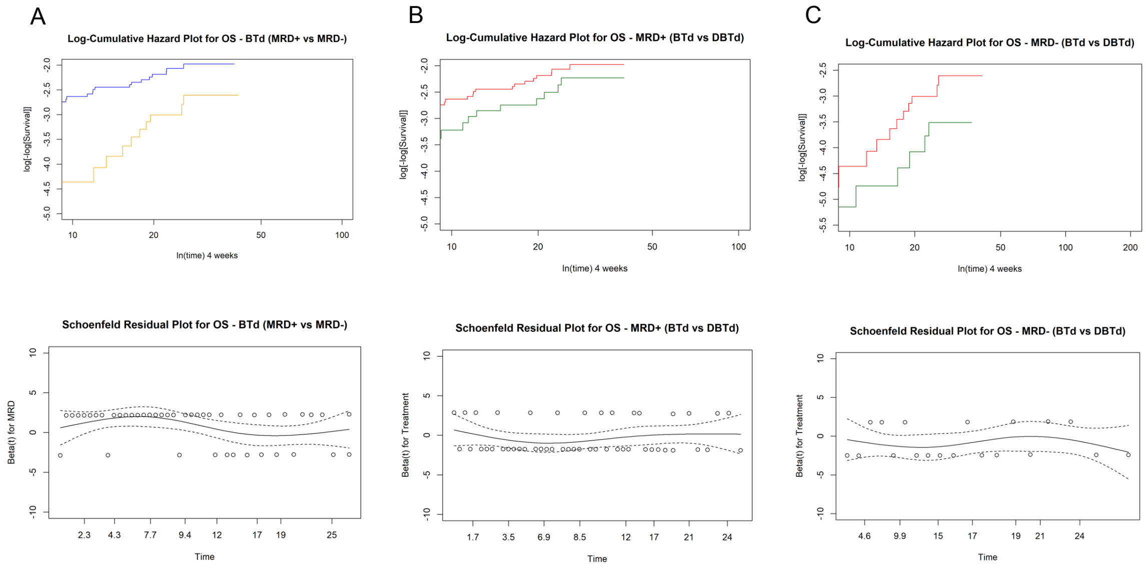

Each panel depicts a log-cumulative hazard plot and Schoenfeld residual plot for OS in each patient population. Panel A compares BTd MRD+ and MRD- patients, panel B compares BTd MRD+ patients and DBTd MRD+ patients, and panel C compares BTd MRD- patients and DBTd MRD- patients.

Abbreviations: BTd, bortezomib, thalidomide, and dexamethasone; DBTd, daratumumab, bortezomib, thalidomide, and dexamethasone; MRD+/-, minimal residual disease positive/negative; OS, overall survival.

Table S2. Average Mean, Median, and Interquartile Ranges Estimated by 2000 Simulations, by Treatment and Model Structure

**Extrapolation Curve**	**Response-based PSM**						**Standard PSM**					
	**DBTd**			**BTd**			**DBTd**			**BTd**		
	**LQ-UQ (IQR)**	**Mean**	**Median**	**LQ-UQ (IQR)**	**Mean**	**Median**	**LQ-UQ (IQR)**	**Mean**	**Median**	**LQ-UQ (IQR)**	**Mean**	**Median**
Exponential	21.28-25.03 (3.75)	22.77	23.38	15.11-17.57 (2.46)	16.30	16.33	23.42-24.77 (1.35)	24.03	24.17	18.72-20.41 (1.69)	19.58	19.58
Weibull	22.54-25.75 (3.21)	23.62	24.50	16.61-20.67 (4.06)	18.32	18.94	15.36-21.58 (6.22)	18.19	18.93	11.72-15.77 (4.05)	14.00	13.53
Log normal	24.35-26.26 (1.91)	25.04	25.53	20.95-22.86 (1.91)	21.71	21.96	22.40-24.47 (2.07)	23.11	23.65	19.13-21.27 (2.14)	20.05	20.30
Log logistic	23.39-26.04 (2.65)	24.33	25.02	18.96-21.65 (2.69)	20.07	20.51	18.79-22.65 (3.86)	20.44	21.01	15.25-18.40 (3.15)	16.73	16.95
Gompertz	25.13-26.43 (1.30)	25.19	25.97	22.99-24.43 (1.44)	22.88	23.88	8.38-20.46 (12.08)	11.13	13.86	7.16-11.39 (4.23)	10.22	8.49
Generalized gamma	24.78-26.36 (1.58)	25.33	25.75	21.43-23.84 (2.41)	22.48	22.83	24.26-25.87 (1.61)	23.46	25.41	16.73-22.09 (5.36)	18.80	20.14

Abbreviations: BTd, bortezomib, thalidomide, and dexamethasone; DBTd, daratumumab, bortezomib, thalidomide, and dexamethasone; IQR, interquartile range; LQ, lower quartile; PSM, partitioned survival model; UQ, upper quartile.
